# Effects of improved home heating on asthma in community dwelling children: randomised controlled trial

**DOI:** 10.1136/bmj.a1411

**Published:** 2008-09-23

**Authors:** Philippa Howden-Chapman, Nevil Pierse, Sarah Nicholls, Julie Gillespie-Bennett, Helen Viggers, Malcolm Cunningham, Robyn Phipps, Mikael Boulic, Pär Fjällström, Sarah Free, Ralph Chapman, Bob Lloyd, Kristin Wickens, David Shields, Michael Baker, Chris Cunningham, Alistair Woodward, Chris Bullen, Julian Crane

**Affiliations:** 1 He Kainga Oranga/Housing and Health Research Programme, University of Otago, Wellington, PO 7343, Wellington South, New Zealand; 2 BRANZ, Porirua City, New Zealand; 3 School of Engineering and Advanced Technology, Massey University, Palmerston North, New Zealand; 4 School of Geography, Environment and Earth Sciences, Victoria University, Wellington; 5 Energy Studies, Physics Department, University of Otago, Dunedin, New Zealand; 6 Wellington Asthma Research Group, University of Otago; 7 Research Centre for Māori Health and Development, Massey University, Wellington; 8 School of Population Health, University of Auckland

## Abstract

**Objective** To assess whether non-polluting, more effective home heating (heat pump, wood pellet burner, flued gas) has a positive effect on the health of children with asthma.

**Design** Randomised controlled trial.

**Setting** Households in five communities in New Zealand.

**Participants** 409 children aged 6-12 years with doctor diagnosed asthma.

**Interventions** Installation of a non-polluting, more effective home heater before winter. The control group received a replacement heater at the end of the trial.

**Main outcome measures** The primary outcome was change in lung function (peak expiratory flow rate and forced expiratory volume in one second, FEV_1_). Secondary outcomes were child reported respiratory tract symptoms and daily use of preventer and reliever drugs. At the end of winter 2005 (baseline) and winter 2006 (follow-up) parents reported their child’s general health, use of health services, overall respiratory health, and housing conditions. Nitrogen dioxide levels were measured monthly for four months and temperatures in the living room and child’s bedroom were recorded hourly.

**Results** Improvements in lung function were not significant (difference in mean FEV_1_ 130.7 ml, 95% confidence interval −20.3 to 281.7). Compared with children in the control group, however, children in the intervention group had 1.80 fewer days off school (95% confidence interval 0.11 to 3.13), 0.40 fewer visits to a doctor for asthma (0.11 to 0.62), and 0.25 fewer visits to a pharmacist for asthma (0.09 to 0.32). Children in the intervention group also had fewer reports of poor health (adjusted odds ratio 0.48, 95% confidence interval 0.31 to 0.74), less sleep disturbed by wheezing (0.55, 0.35 to 0.85), less dry cough at night (0.52, 0.32 to 0.83), and reduced scores for lower respiratory tract symptoms (0.77, 0.73 to 0.81) than children in the control group. The intervention was associated with a mean temperature rise in the living room of 1.10°C (95% confidence interval 0.54°C to 1.64°C) and in the child’s bedroom of 0.57°C (0.05°C to 1.08°C). Lower levels of nitrogen dioxide were measured in the living rooms of the intervention households than in those of the control households (geometric mean 8.5 μg/m^3^ *v* 15.7 μg/m^3^, P<0.001). A similar effect was found in the children’s bedrooms (7.3 μg/m^3^ *v* 10.9 μg/m^3^, P<0.001).

**Conclusion** Installing non-polluting, more effective heating in the homes of children with asthma did not significantly improve lung function but did significantly reduce symptoms of asthma, days off school, healthcare utilisation, and visits to a pharmacist.

**Trial registration** Clinical Trials NCT00489762.

## Introduction

Asthma is one of the most prevalent chronic diseases in childhood. In New Zealand about 25% of children report symptoms of asthma, and asthma is the second most common reason for children being admitted to hospital.^1^ As well as the stress associated with having a chronic disease, asthma can lead to higher utilisation of health services and drug costs.^2^ Children with asthma are likely to have more days off school, with adverse effects on academic performance,^3^ and their caregivers may lose significant time from work.^4^

Evidence is growing that symptoms of asthma can be aggravated or triggered by adverse aspects of the indoor environment.^5^ ^6^ Evidence from studies of excess morbidity and mortality during winter in temperate climates shows that temperatures in many homes are below the levels recommended by the World Health Organization for maintaining health in vulnerable populations.^7^ This is the case in New Zealand, where home heating seems not to be treated as a necessity like it is in the cooler parts of continental Europe.^7^ ^8^ ^9^

As well as cold temperatures, factors such as damp, mould, and pollutants (for example, volatile organic compounds and combustion byproducts from heating and second hand smoke) have been implicated in aggravating the symptoms of asthma.^10^ The relative importance of these factors together with house dust mites in causing asthma and aggravating symptoms such as cough and wheeze is difficult to establish and the pathophysiology remains unclear.^11^ ^12^

Infants and elderly people spend proportionally more time indoors^13^ and children may be especially vulnerable to indoor air pollutants that affect development and lung function because of their immature immune systems and rapid growth and development.^14^ Infants and children also inhale a larger dose of air per unit of body mass at a given level of activity and therefore more pollutants than do adults in the same environment.^15^

Nitrogen dioxide has been widely used as a marker for outdoor air quality in studies of pollution related to vehicle emissions, but the most important source of personal exposure to nitrogen dioxide is unflued gas appliances.^16^ ^17^ ^18^ Pollutants emitted indoors have about a 1000-fold greater chance of being inhaled than those emitted from proximate outdoor sources.^19^ ^20^ ^21^ In New Zealand a third of households have unflued gas heaters^22^ and previous studies have found that rooms with such heaters have higher concentrations of nitrogen dioxide than rooms with electric or flued gas heaters.^23^

Nitrogen dioxide is a proinflammatory gas and can exacerbate respiratory symptoms such as wheeze or cough. It can reduce immunity to lung infections and increase the severity and duration of an episode of flu.^24^ Several cross sectional studies have investigated the impact of home heating on morbidity, mostly respiratory symptoms in children.^25^ ^26^ Reports on the impact of cooking by gas on asthma have conflicted; some studies have shown a twofold increase in prevalence of asthma among households that cook by gas,^27^ ^28^ whereas others have found no difference.^29^ ^30^ Some studies have also established a link between type of heating and respiratory symptoms in children.^31^ Unflued gas heaters and cookers release water vapour during the combustion process and may partly explain the link between dampness and symptoms of asthma.^32^

Relatively few intervention studies have examined the effects of housing improvements on health and fewer still have shown an impact on symptoms of asthma.^33^ ^34^ One study highlighted the difficulty of implementing community based intervention trials for controlling asthma.^34^ An intervention study that involved removing unflued gas heaters from school classrooms found a significant reduction in children’s self reported asthma symptoms.^35^ A before and after study of an intervention to reduce damp in the homes of children with asthma found a significant and cost effective reduction in respiratory symptoms, particularly cough at night and the number of days off school.^36^ ^37^

We previously carried out a randomised controlled trial of the effects on respiratory health of insulating homes.^38^ Although we did not look exclusively at people with asthma the results indicated that even basic housing interventions, such as retrofitting insulation, can raise indoor temperatures and improve respiratory symptoms.^39^ During the analysis of this study we became concerned about the number of children with asthma living in homes with ineffective heating. The use of ineffective and polluting heating systems in many New Zealand houses suggested that improved heating might provide further health gains over and above that of insulation. We investigated the impact of a heating intervention on symptoms of asthma in children in homes that had been insulated before this trial.

## Methods

We carried out a randomised controlled trial in which baseline measures were collected in winter (June to September) 2005. Households in the intervention group were allocated a non-polluting, more effective replacement heater (heat pump, wood pellet burner, flued gas) before the follow-up winter of 2006. The control group received a similar heater in 2007, after the final data collection in 2006.

The study was carried out in five areas in New Zealand—Porirua and the Hutt Valley in the North Island and Christchurch, Dunedin, and Bluff in the South Island. Households were recruited from December 2004 to May 2005. We identified community coordinators in nine local asthma societies and primary health organisations (five with close ties to Maori, indigenous New Zealanders who have a greater burden of respiratory illness). The community coordinators invited families who had a child with asthma to participate in the study. The study was also publicised in radio interviews. Overall, 899 applications were received of which 422 (47%) met the inclusion criteria: the family lived in a study area and had a child aged between 6 and 12 years with doctor diagnosed asthma and symptoms in the past 12 months; the child slept at least four nights a week in the house; the house contained a less effective form of heating (unflued gas or plug-in electric heaters); the family intended to live in the house over the two winter periods; and the homeowner agreed that the household could take part in the study. If more than one child in a household met the study criteria then the child whose birthday occurred first after 1 June became the index child.

After enrolment and before winter 2005 participating houses were insulated to the current New Zealand building code standard, as our previous study had shown positive effects from insulation alone.^39^ This insulation was considered necessary to achieve consistency of the thermal envelope between the participating houses.

### Baseline outcome measures

The research team trained the community coordinators in the informed consent procedures, completion of questionnaires and symptom diaries, use of Piko meters (nSpire Health, Longmont, CO) for measuring peak expiratory flow rate and forced expiratory volume in one second (FEV_1_), and correct placement of temperature loggers and nitrogen dioxide diffusion tubes. At the end of winter 2005 the community coordinators assisted households in completing the questionnaires. To check for reporting bias we included three outcomes for which we hypothesised no association with the intervention: diarrhoea, vomiting, and ear infections. The questionnaires are available at www.wnmeds.ac.nz/healthyhousing.html.

Children kept daily diaries of their respiratory symptoms, giving a score from 0 to 3 for each of six lower respiratory tract symptoms and each of five upper respiratory tract symptoms, as used in a previous study.^24^ They also recorded the number of puffs of asthma preventer and reliever drugs daily and whether a reliever was used at night. The community coordinators downloaded data from the Piko meters on to laptops at each of four monthly visits. The diffusion tubes for recording nitrogen dioxide levels were placed in a safe, standard location in the living room and child’s bedroom (1.8 m from the ground and 5 cm from the wall). Temperature loggers, positioned on the diffusion tube holders, recorded the temperature every 20 minutes. The diffusion tubes and temperature loggers were changed at each visit.

### Intervention

The advantages and disadvantages of the three heater types (heat pump, wood pellet burner, or flued gas) were explained at community meetings and described on the research group’s website. The homeowners chose a replacement for their existing heaters. Landlords were encouraged to consider their tenants’ preferences. An independent statistician then randomised the households to intervention or control groups, stratified by area and heater choice. The heaters were installed in the intervention houses before winter 2006. The control group were told that they would receive a replacement heater at the end of the study.

### Outcome measurements

Baseline measures were repeated after the intervention at follow-up in winter 2006. In 2006 we changed the format of the questionnaire, eliminating the instruction that if people answered no to the question on wheeze they were to skip related questions, and we added a question on twisted ankles to check for reporting bias. We changed the method of recording the data from the Piko meter to recording readings in the symptom diary, as the downloaded data from 2005 were unsatisfactory. The temperature loggers were reprogrammed to record temperature every hour instead of every 20 minutes and to remain in place for the whole winter. We re-emphasised to the community coordinators the need for accuracy and timely retrieval of data.

Participants signed informed consent forms. Parents signed on behalf of their children. Households were told that the heaters were the property of the homeowner.

### Outcomes and sample size

The primary outcome measure was changes to lung function. The study was powered to show a reduction in the amplitude of diurnal changes, expressed as a percentage of their mean peak expiratory flow rate over winter (amplitude % mean). A total of 430 children were required to detect a 15% reduction in the intervention group compared with the control group, assuming a correlation with the previous year’s measurement of 0.4, with 90% power, α at 0.05, and a 15% non-response rate. Secondary outcomes were reported asthma symptoms, scores for lower respiratory tract symptoms from the diaries, asthma drug use, healthcare utilisation, and days off school. Intermediate outcomes were temperature and nitrogen dioxide levels in the living room and child’s bedroom.

### Statistical analysis

Epi-Info version 3.4.1 (Centers for Disease Control and Prevention, Atlanta, GA) was used to double enter the responses to the questionnaire and data from the diaries. We cleaned and analysed the data using the statistical package R version 2.4.1 (www.r-project.org/). The binary information (for example, dry cough at night yes or no) was analysed using both standard generalised linear models and analysis of covariance (adjusting for outcome at baseline) generalised linear models with the logistic link function. From these models we derived unadjusted odds ratios (95% confidence intervals) as measures of effect size and adjusted odds ratios (95% confidence intervals) as measures for precision of estimates. We similarly analysed the numerical counts (for example, number of days off school) but with a Poisson link function. For these models we present the change for the intervention group compared with the control group as mean number of events, with 95% confidence intervals.

We present data only for the index child, eliminating the need to control for clustering by household. When baseline measurements were not available (0% to 2% of participants for questionnaires, 24% for symptom diaries, and 33% for lung function measures), we used the overall mean and validated the results using the complete data. Results for the interpolated data were predominantly within 5% of the results for the non-interpolated data.

In the daily diaries individual respiratory symptoms were recorded on a nominal scale (0 to 3) as used previously.^24^ We combined the results for the three questions on cough and the three on wheeze (morning, day, and night) to give a score for cough and a score for wheeze, each on a scale of 0 to 9. These six questions on lower respiratory tract symptoms were also combined to give a score on a scale of 0 to 18, and the five questions on upper respiratory tract symptoms were likewise combined to give a score on a scale of 0 to 15. We measured FEV_1_ and peak expiratory flow rate from three good forced expiratory manoeuvres, measured each morning and evening. The three outcomes presented are the best blow in the morning, in the evening, and that day.

For all daily records we used a linear mixed model^40^ (lmer function in R lme4 library). This model uses two levels: in the first level (intraindividual) each child’s sequence of daily responses is modelled by an intercept term, on the next level (interindividual) the intervention is then evaluated for its effect on this intercept term. The model finds the estimate of effect size that maximises the model likelihood as given by a distribution function. We used a Poisson distribution to model the daily symptom scores and their combinations and the number of puffs for preventer and reliever. A normal distribution function was used for peak expiratory flow rate and FEV_1_.

We tested model distributions by examining the dispersion variable in the Poisson models and QQ plots for the normal models. Ethnicity was collected using the standardised self identity question of Statistics New Zealand. When multiple ethnicities were reported we used prioritised Māori and then Pacific ethnicities.^41^

## Results

Overall, 409 households were randomised. After exclusions and withdrawals 349 (85%) households remained (figure[Fig fig1]). After randomisation no significant differences were found between intervention and control groups for all relevant measured variables (table 1). Of households that completed the study, 175 heaters were installed in the intervention group (131 heat pumps, 39 wood pellet burners, and five flued gas heaters). The three intervention households that withdrew from the study had chosen heat pumps.

**Figure fig1:**
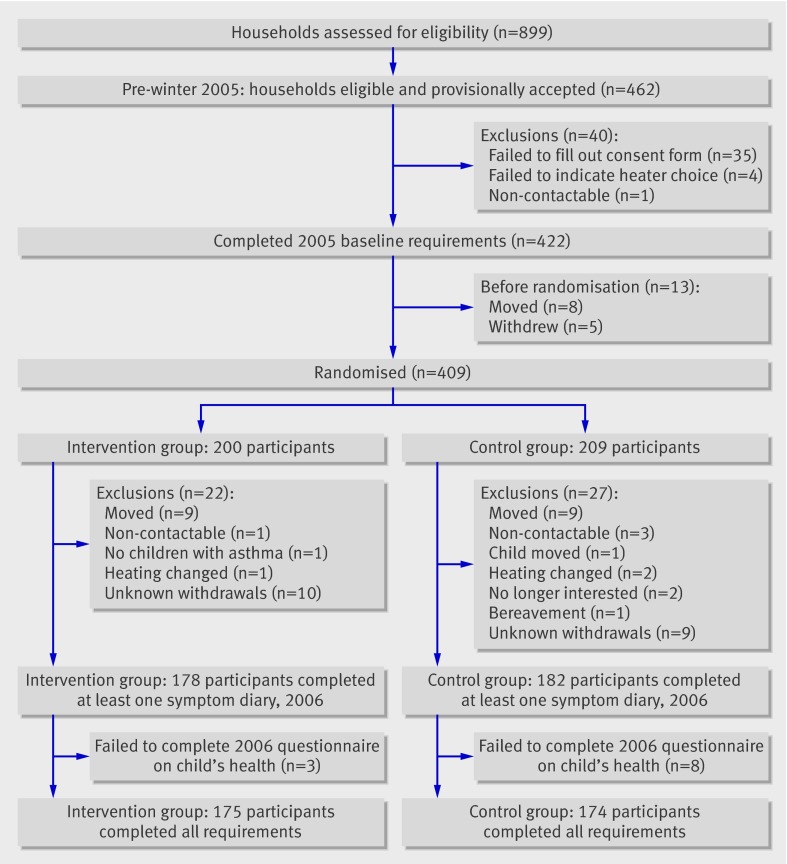
Flow of households and children through study

**Table 1 tbl1:** Comparison between children with asthma in intervention and control groups. Values are percentages (numbers) unless stated otherwise

Variables	Intervention group (n=175)	Control group (n=174)	Mean difference (95% CI)
Boys	54.29 (95)	60.34 (105)	−6.05 (−16.99 to 4.87)
Mean (No) age (years)	10.06 (175)	10.02 (174)	0.04 (−0.23 to 0.30)
Ethnicity:			
Māori	34.86 (61)	37.36 (65)	−2.50 (−13.15 to 8.15)
Pacific peoples	13.14 (23)	7.47 (13)	5.67 (−0.01 to 12.60)
Other	52.00 (91)	43.68 (76)	8.32 (−2.70 to 19.34)
Family history of asthma	53.71 (94)	54.02 (94)	−0.31 (−11.08 to 10.46)
Smoker in home	20.00 (35)	22.41 (39)	−2.41 (−6.7 to 11.56)
Region:			
Porirua	21.71 (38)	22.41 (39)	−0.7 (−9.97 to 8.57)
Hutt Valley	33.71 (59)	28.74 (50)	4.97 (−5.30 to 15.26)
Christchurch	27.43 (48)	28.57 (50)	−1.31 (−11.31 to 8.69)
Dunedin and Bluff	17.14 (30)	20.11 (35)	−2.97 (−11.71 to 5.77)
Gas heating before study	55.43 (97)	59.20 (103)	3.77 (−7.18 to 14.71)

Table 2 shows the results for the lung function tests done in the morning and evening. The β coefficients are the average daily change in best blow for FEV_1_ and peak expiratory flow rate for the intervention group compared with the control group. In 2006, after the intervention, a non-significant increase occurred in daily FEV_1_ (129.4 ml, 95% confidence interval −21.4 to 280.3; P=0.09) and daily peak expiratory flow rate (12.12 l/min, 95% confidence interval −4.76 to 29.00; P=0.16).

**Table 2 tbl2:** Effect of intervention on lung function in children

Variable	No of person days	No of children	Unadjusted			Adjusted*	
			Effect size (β) (95% CI)	P value		Effect size (β) (95% CI)	P value
Amplitude % mean peak expiratory flow rate	26 439	359	1.41 (−2.80 to 5.62)	0.51		1.41 (−2.80 to 5.62)	0.62
Daily FEV_1_	26 960	360	130.7 (−20.3 to 281.7)	0.09		129.4 (−21.4 to 280.3)	0.09
Morning FEV_1_	22 157	346	57.6 (−74.8 to 190.1)	0.40		57.0 (−75.4 to 189.4)	0.4
Evening FEV_1_	23 406	353	121.7 (−37.0 to 280.3)	0.13		120.6 (−38.1 to 279.4)	0.14
Daily peak expiratory flow rate	27 007	360	12.29 (−4.57 to 29.15)	0.15		12.12 (−4.76 to 29.00)	0.16
Morning peak expiratory flow rate	22 450	347	9.01 (−7.54 to 25.56)	0.28		8.92 (−7.66 to 25.50)	0.29
Evening peak expiratory flow rate	23 413	353	12.30 (−4.14 to 28.74)	0.14		12.17 (−4.3 to 28.63)	0.15
Percentage predicted†:							
FEV_1_	22111	283	2.46 (−11.62 to 16.54)	0.73		2.6 (−11.52 to 16.73)	0.72
Morning FEV_1_	18 465	276	−1.07 (−12.62 to 10.49)	0.85		−0.87 (−12.46 to 10.72)	0.88
Evening FEV_1_	19 279	280	2.60 (−12.00 to 17.20)	0.73		2.59 (−12.05 to 17.24)	0.73
Peak expiratory flow rate	22 139	283	3.54 (−1.41 to 8.48)	0.16		3.56 (−1.39 to 8.52)	0.16
Morning peak expiratory flow rate	18 613	276	2.99 (−1.77 to 7.75)	0.22		3.01 (−1.77 to 7.78)	0.22
Evening peak expiratory flow rate	19 279	280	3.65 (−1.14 to 8.45)	0.14		3.68 (−1.13 to 8.49)	0.13

FEV_1_=forced expiratory volume in one second.

ß coefficients are estimated effect size of intervention on peak expiratory flow rate and FEV_1_.

*Controlled for baseline measure.

†Percentage of predicted FEV_1_ or peak expiratory flow rate based on 283 available heights.

Table 3 shows self reported health status and the outcomes used to test for reporting bias. A statistically significant improvement occurred in half the symptoms—that is, fewer children in the intervention group than control group had health rated as suboptimal (poor, fair *v* good, very good, excellent) by their parents (adjusted odds ratio 0.48, 95% confidence interval 0.31 to 0.74; P<0.001). Parental reports showed a reduction in sleep disturbed by wheeze (adjusted odds ratio 0.55, 95% confidence interval 0.35 to 0.85; P<0.001) and dry cough at night (0.52, 0.32 to 0.83; P=0.01).The other three symptoms (attacks of wheezing, speech limited by wheeze, and wheeze during exercise) improved but not significantly. Of the four outcomes used to test for reporting bias (diarrhoea, vomiting, ear infections, and twisted ankles) only twisted ankles showed a significant effect.

**Table 3 tbl3:** Effect of heating intervention on parent reported health outcomes in children

Health outcome	No of children (n=349)	% with outcome in control group	Unadjusted			Adjusted*	
			Odds ratio (95% CI)	P value		Odds ratio (95% CI)	P value
Poor or fair health†	346	60	0.46 (0.30 to 0.71)	<0.001		0.48 (0.31 to 0.74)	<0.001
Attacks of wheezing‡	345	43	0.68 (0.44 to 1.05)	0.08		0.71 (0.45 to 1.11)	0.13
Sleep disturbed by wheeze	344	60	0.54 (0.35 to 0.83)	0.005		0.55 (0.35 to 0.85)	<0.001
Speech limited by wheeze	344	19	0.74 (0.43 to 1.27)	0.27		0.69 (0.40 to 1.18)	0.18
Wheeze during exercise	344	66	0.73 (0.46 to 1.14)	0.16		0.67 (0.42 to 1.06)	0.09
Dry cough at night	345	66	0.50 (0.32 to 0.79)	0.003		0.52 (0.32 to 0.83)	0.01
Diarrhoea	343	34	0.81 (0.51 to 1.26)	0.34		0.72 (0.45 to 1.16)	0.18
Vomiting	344	31	1.01 (0.64 to 1.59)	0.98		0.88 (0.55 to 1.40)	0.58
Ear infections	344	24	1.40 (0.85 to 2.31)	0.19		1.16 (0.68 to 1.99)	0.58
Twisted ankles	346	12	1.86 (1.03 to 3.35)	0.04		—§	—§

*Controlled for baseline measure.

†Compared with good, very good, and excellent health.

‡More than four attacks per week compared with fewer than three attacks.

§Question not asked in 2005.

Results from the daily symptom diaries are presented as mean ratios—the average score for the intervention group divided by the average score for the control group (table 4). The results were consistent with those of the questionnaires completed at the end of winter, by showing a significant reduction in lower respiratory tract symptoms (adjusted mean ratio 0.77, 95% confidence interval 0.73 to 0.81; P=0.013), cough (0.75, 0.62 to 0.92; P=0.005), and wheeze (0.67, 0.50 to 0.91; P=0.011). A non-significant reduction occurred in the use of reliever during the night (adjusted odds ratio 0.55, 95% confidence interval 0.28 to 1.08; P=0.081).

**Table 4 tbl4:** Effect of heating intervention on daily differences of asthma symptoms and drug use as reported in daily diaries

Variable	No of person days	No of children	Unadjusted			Adjusted*	
			Mean ratio† (95% CI)	P value		Mean ratio† (95% CI)	P value
Lower respiratory tract symptoms	23 475	345	0.83 (0.66 to 1.05)	0.12		0.77 (0.73 to 0.81)	0.01
Cough at night	26 532	352	0.80 (0.63 to 1.00)	0.05		0.72 (0.59 to 0.89)	0.002
Wheeze at night	26 407	351	0.78 (0.54 to 1.12)	0.18		0.67 (0.49 to 0.93)	0.02
Cough on waking	26 514	352	0.74 (0.58 to 0.94)	0.02		0.67 (0.53 to 0.84)	<0.001
Wheeze on waking	26 417	351	0.68 (0.49 to 0.94)	0.02		0.60 (0.45 to 0.81)	0.001
Cough during day	27 348	365	0.90 (0.75 to 1.10)	0.31		0.84 (0.70 to 1.01)	0.06
Wheeze during day	27 117	363	0.85 (0.61 to 1.17)	0.32		0.78 (0.59 to 1.04)	0.09
Cough symptoms	23 713	349	0.82 (0.67 to 1.02)	0.08		0.75 (0.62 to 0.92)	0.005
Overall wheeze symptoms	23 532	345	0.76 (0.54 to 1.07)	0.11		0.67 (0.50 to 0.91)	0.01
No of reliever puffs	27 261	364	0.73 (0.46 to 1.14)	0.17		0.68 (0.44 to 1.05)	0.08
Reliever use at night (yes or no)‡	26 725	352	0.52 (0.24 to 1.13)	0.10		0.55 (0.28 to 1.08)	0.08
No of preventer puffs	27 567	363	1.05 (0.61 to 1.8)	0.87		1.08 (0.67 to 1.74)	0.74
Upper respiratory tract symptoms	26 844	360	0.95 (0.76 to 1.19)	0.65		0.92 (0.74 to 1.14)	0.43

*Adjusted for baseline outcome.

†Average score for intervention group divided by average score for control group.

‡Binary model used and results presented as odds ratio.

Parents of children in the intervention group reported fewer days off school for asthma during the winter of 2006: 0.73 (95% confidence interval −0.67 to 1.94, P=0.28; see table 5). Statutory school records on absence, however, showed that children in the intervention group had 1.80 fewer days off school (0.11 to 3.13, P=0.04) during the winter term (100 school days).

**Table 5 tbl5:** Effect of heating intervention on parental reports of healthcare utilisation and days off school

Variable	No of children (n=349)	Mean occurrence per child in control group	Unadjusted			Adjusted*	
			Reduction due to intervention (95% CI)	P value		Reduction due to intervention (95% CI)	P value
Days off school (school records)	267	9.47	1.55 (0.33 to 3.12)	0.10		1.80 (0.11 to 3.13)	0.04
Days off school (self report)	333	6.67	0.93 (−0.67 to 2.2)	0.23		0.73 (−0.67 to 1.94)	0.28
Visits to doctor for asthma	323	1.33	0.31 (−0.04 to 0.57)	0.07		0.40 (0.11 to 0.62)	0.01
Episodes of colds or flu	341	2.39	0.50 (0.14 to 0.79)	0.01		0.50 (0.14 to 0.79)	0.01
Other visits to doctor	333	1.08	0.24 (−0.05 to 0.45)	0.10		0.27 (0.01 to 0.46)	0.04
Visits to nurse for asthma	335	0.38	0.07 (−0.22 to 0.22)	0.54		0.05 (−0.24 to 0.2)	0.67
Visits to pharmacist for asthma	345	0.37	0.26 (0.1 to 0.32)	0.01		0.25 (0.09 to 0.32)	0.01
Visits to after hours clinic for asthma	348	0.12	0.01 (−0.12 to 0.07)	0.90		0.00 (−0.12 to 0.07)	0.92
Other visits to after hours clinic	345	0.16	0.07 (−0.08 to 0.12)	0.26		0.07 (−0.07 to 0.13)	0.22

*Controlled for baseline measure.

Parents of children in the intervention group also reported an average of 0.5 fewer episodes of cold and flu (95% confidence interval 0.14 to 0.79, P=0.01), 0.4 fewer visits to the doctor for asthma (0.11 to 0.62, P=0.01), 0.25 fewer visits to a pharmacist for asthma (0.09 to 0.32, P=0.01), and 0.27 fewer visits to the doctor for non-asthma related conditions (0.01 to 0.46, P=0.04).

### Exposure

After the intervention, during the winter of 2006, the average living room temperature of intervention households was 17.07°C compared with 15.97°C for control households: a difference of 1.10°C (95% confidence interval 0.54°C to 1.67°C, P<0.001). Similarly, the average temperature in the child’s bedroom for intervention households was 14.84°C compared with 14.26°C for control households: a mean difference of 0.57°C (95% confidence interval 0.05°C to 1.08°C, P=0.03).

Exposure to low temperatures, measured as degree hours—that is, hours per day, multiplied by number of degrees less than 10°C—was over 50% less in the intervention group than control group. In the living room the exposure was 1.13 degree hours in the intervention group compared with 2.31 degree hours in the control group, a difference of 1.18 degree hours (95% confidence interval 0.49 to 1.93, P=0.001). Exposure in the child’s bedroom was 2.03 degree hours in the intervention group compared with 4.29 degree hours in the control group, a difference of 2.26 degree hours (0.99 to 2.34, P<0.001).

Ambient outdoor nitrogen dioxide levels were the same in both groups, but indoor levels were significantly reduced in the intervention group. In 2006 the intervention group had significantly (P<0.001) lower geometric mean nitrogen dioxide levels in the living room than the control group (8.5 μg/m^3^ *v* 15.7 μg/m^3^, P<0.001). A similar significant effect was found in the child’s bedroom (7.3 μg/m^3^ *v* 10.9 μg/m^3^, P<0.001).^42^

## Discussion

Installing non-polluting, more effective home heating in the households of children with asthma in New Zealand did not significantly improve lung function but did lead to a reduction in symptoms of asthma, improved wellbeing, and fewer days off school.

The housing, heating, and health study was a community based trial on the impact of improved heating on the health of children with asthma. The study received funding from both the public and the private sector. We used a partnership model that entailed working with 18 different community agencies and private organisations and we were able to maintain a high level of support throughout. This study also showed the success of a treaty based partnership research design, which saw involvement and responsibility of Maori at all levels of the research thus facilitating the participation of Maori community organisations and participants.

We used lung function as the primary outcome measure because it can be objectively recorded. We did not, however, find a statistically significant improvement in either FEV_1_ or peak expiratory flow rate. One possible reason for failing to achieve statistical significance is that the improvement in symptoms led to less use of reliever drugs and this introduced a bias that reduced the observed effect on lung function. Another possibility is an error in our measurement of lung function; the Piko meter proved susceptible to unstable readings with poor technique. In the second year we were aware of this limitation and the community workers took greater care to check the children’s technique at each visit. Problems of this type reduce statistical power but are unlikely to change the direction of the result because of the randomised nature of the study design. Another possible reason is that the magnitude of the effect from the intervention was smaller then anticipated in our power calculations. A possibility remains that no effect exists.

Although improvement in lung function is considered ideal for assessing control of asthma, little correlation exists between measures of airway calibre and symptoms of asthma,^43^ nor is there good agreement between asthma severity as reported by a doctor or as determined by lung function.^44^ Indeed, questionnaires to determine the control of asthma have been shown to be better discriminators of control than diaries of peak flow measurements.^45^

Although we were unable to show significant improvements in lung function, the direction of almost all the results was towards an improvement in health. We found significant reductions in asthma symptoms and time lost from school and reductions in dry cough at night and sleep disturbed by wheeze. These reductions in nocturnal symptoms were supported by similar findings from the children’s daily symptom diaries. These changes are consistent with significantly reduced exposure to nitrogen dioxide at home in the evening and at night. The use of reliever drugs was also reduced at night and during the day among children in the intervention group compared with children in the control group, although this also failed to reach statistical significance.

Parents reported fewer lower respiratory tract symptoms in children in the intervention group as well as fewer absences from school and visits to the doctor or a pharmacist for asthma, colds, and flu.. The greater magnitude of changes in these symptoms and behaviours compared with measured lung function suggests that the symptoms associated with exposure to nitrogen dioxide may have been predominantly of an irritant nature rather than inducing changes in airway calibre.

The results of this study build on earlier research indicating that higher indoor temperatures and lower nitrogen dioxide delivered by improved heating are associated with reduced asthma symptoms^37^ and our own work showing that retrofitting insulation in homes improves health status and respiratory symptoms.^39^ Over and above the improvements in health already obtained from insulating these houses, our results show the impact of less polluting, more effective heating on children’s self reported severity of asthma symptoms. The reduction of symptoms is reflected in fewer days off school and fewer visits to the doctor.

Although only half the houses in each group had unflued gas heaters at baseline, the large reduction in nitrogen dioxide levels in the intervention group suggests a dual benefit of the new heaters: raising the indoor temperature and reducing nitrogen dioxide levels. Further analyses of nitrogen dioxide measurements and the use of the new heaters are being undertaken for a subsample of 69 intensively monitored houses. In addition we are analysing the health records of other family members to see if the intervention had an impact on other dimensions and objective measures of health, and we are carrying out a cost benefit analysis. The capital cost of the study intervention was not borne by the participants and was relatively high for a population based intervention ($NZ3000; £1137; €1392; $2007 average per house) compared with the average cost of the unflued gas or portable electric heater ($NZ100 per house).

The major limitation in randomised trials of this sort is the impossibility of implementing blinding of the participants or field workers, once they visited the home. This could have resulted in a reporting bias, although as all participants knew at the outset of the study that they would receive new heaters it is unclear in which direction this would be. However, the outcome measurements included both self report and blinded measures and when both were available, as with days off school, the blinded measures showed a greater effect, suggesting any reporting bias was in the direction of minimising the intervention effect. In addition, three of the four questions that tested for reporting bias were not significantly affected by the intervention. The other outcome (twisted ankles) showed increased reporting in the intervention group, also suggesting that any bias was in the direction of minimising the effect of the intervention.

Another important issue is the choice of primary outcome measure; we chose lung function because it was more objective and because the study could not be double blinded. It may be that lung function is less important to the daily life of children with asthma than are the frequency and severity of symptoms. In addition symptoms may be more sensitive to change and more reliable than laboratory based measures carried out by children in a community setting.

As highlighted by one study, managing a community trial with community organisations as partners is challenging for all parties.^34^ For example, many people did not know initially whether their house was insulated or what kind of heating they had, so the visits by community coordinators before the study to establish whether participants met the inclusion criteria were more challenging and expensive than anticipated. The study had some methodological difficulties; in particular during the 2005 baseline year the Piko meters did not perform as well as we had expected. They proved fragile, were prone to recording high readings with misuse, and the data were difficult to download. In 2006 we amended the study protocol by asking the children to write each measurement from the Piko meter in the symptom diary and encouraged the community workers to check these carefully to ensure compliance and technique. To remind the children to use their meters and diaries we gave them digital alarm clocks and fridge magnets and stickers with cartoon characters, which worked well. On the final visit we gave each child a certificate of appreciation.

The results suggest that improving both the type and amount of heating in the homes of children with asthma does not significantly affect measured lung function but does have several beneficial effects. Houses in this study included all forms of tenure, and the households had a range of incomes, so apportioning the relative benefits to the participants and to the public good is an important policy issue. In the United Kingdom, Scotland has made the policy decision that installing central heating in all social and pensioner housing, regardless of tenure, is largely for the good of the public and will pay more health dividends than focusing on clinical waiting lists, as is done in England.^46^ The heaters used in this study were non-polluting and more environmentally sustainable than the less effective heaters that were replaced, an additional public good benefit in terms of mitigating climate change.^47^

### Conclusion

Although the improvement in lung function was not statistically significant, large improvements were found in the symptoms of asthma. These patient centred measures are arguably as important to the daily life of children with asthma as more objective measures, and adopting this environmental intervention is an effective adjunct to the pharmaceutical treatment of asthma symptoms.

**What is already known on this topic**

Observational, but few intervention, studies have shown associations between asthma symptoms and dampness and cold in poorly heated homes

**What this study adds**

Non-polluting, effective heating did not significantly affect measured lung function of children with asthma but it improved wellbeing and reduced symptoms of asthma and days off school

## Notes

We thank the children and their families; our community partners in Porirua (Porirua Health Plus), in the Hutt Valley (Tu Kotahi Māori Asthma Trust, Community Planners, and Mid-Valley PHO, and Waiwhetu Marae), in Christchurch (Community and Public Health and Pacific Trust Canterbury), in Dunedin (Otago Asthma Society), and in Bluff (Te Runaka o Awarua) and in 2005 Te Ropu Awhina (Te Amorangi Richmond), Jo-Ani Robinson, and Paul Leisham; Richard Edwards for his comments on this paper; James Findlater for his graphics; Smartpower for managing the tenders and contracts as well as the retrofit teams; and the primary health organisations, schools, and power companies who supplied us with records. A full list is available on www.wnmeds.ac.nz/healthyhousing.html.

Contributors: PH-C raised funds, carried out the study, interpreted the results, wrote the paper, and approved the final version. She is the guarantor. NP carried out the study, analysed the data, and critically interpreted the results and drafts. PH-C, JC, RC, AW, and HV conceptualised and designed the study and along with all authors participated in the analysis and interpretation of the data, revised the manuscript critically for important intellectual content, and approved the version to be published.

Ethical approval: This study was approved by the ethical committees of all participating centres.

Provenance and peer review: Not commissioned; externally peer reviewed.

## Notes

**Cite this as:** *BMJ* 2008;337:a1411
